# Prevalence of primary headaches in Germany: results of the German Headache Consortium Study

**DOI:** 10.1007/s10194-012-0425-x

**Published:** 2012-03-07

**Authors:** M-S Yoon, Z. Katsarava, M. Obermann, G. Fritsche, M. Oezyurt, K. Kaesewinkel, A. Katsarova, I. Santowski, H. Diener, S. Moebus

**Affiliations:** 1 Department of Neurology, Ruhr-University Bochum, Gudrunstr. 56, 44791 Bochum, Germany; 2 Department of Neurology and Headache Center, University Duisburg-Essen, Hufelandstr. 55, 45147 Essen, Germany; 3 Institute for Medical Informatics, Biometry and Epidemiology, University Duisburg-Essen, Hufelandstr. 55, 45122 Essen, Germany

**Keywords:** Migraine, Tension-type headache, Chronic daily headache, Prevalence

## Abstract

We investigated the prevalence of migraine (MIG), tension-type headache (TTH), and chronic headache in a population-based sample in Germany. A total of 18,000 subjects aged between 18 and 65 years were screened from 2003 until 2005 using a validated questionnaire. Overall 9,944 participants (55.2%) responded (mean age 43 ± 13.1 years, 52.7% women). Headache frequency <15 days/month was reported by 5,350 (55.5%) subjects of whom 1,601 (16.6%, [95% confidence interval (95% CI): 15.9–17.4]) reported episodic MIG, 1,202 (12.5%, 95% CI 11.8–13.1) episodic TTH, and 1,150 (11.9%, [11.3–12.6]) episodic MIG + episodic TTH, 1,396 (14.5%, [13.8–15.2]) unclassifiable headache. In women, episodic MIG peaked between 36 and 40 years, episodic MIG + TTH between 18 and 35 years and episodic TTH between 56 and 66 years. In men, episodic MIG was predominant between 36 and 45 years, episodic MIG + TTH between 26 and 35 years and episodic TTH showed comparable frequency between 36 and 66 years. Headache ≥15 days/month was reported by 2.6% (*n* = 255, [95% CI 2.3–3]). Chronic MIG was reported by 1.1% (*n* = 108, [0.91–1.33]), chronic TTH (*n* = 50, [95% CI 0.4–0.7]), chronic MIG + TTH 0.8% (*n* = 74, 95% CI 0.6–0.9) and unclassifiable headache 0.2% (*n* = 23, [95% CI 0.1–0.3]). Chronic headache was more frequent in women compared to men with the highest prevalence between 46 and 65 years. It is of note that the number of subjects with chronic headache is small in all age groups. The results of our large, population-based study provide reliable, age- and sex-specific estimates of the prevalence of primary headache disorders in Germany. The prevalence with respect to episodic and chronic primary headache disorders in Germany is comparable to other European countries and the USA.

## Introduction

The two most common primary headache disorders, migraine (MIG) and tension-type headache (TTH), affect up to 80% of the general population worldwide [1]. Both disorders are classified by the International Headache Society (IHS) into two different categories depending on headache frequency: episodic (<15 headache days per month) and chronic (≥15 headache days per month) [2]. Chronic daily headache [including both, chronic migraine (cMIG) and chronic TTH (cTTH)] is quite common and affects approximately 3–4% of the general population [3–5]. In particular, cMIG is associated with a significant burden of illness for individuals, their families and the society [6, 7]. However, comprehensive data of the prevalence in the general population with respect to age and sex are rare. The German Headache Consortium Study (GHC) is an ongoing population-based, longitudinal cohort study designed to investigate the prevalence and incidence of primary headache disorders in the general population of Germany. Here, we present detailed baseline data on the prevalence of MIG, TTH and chronic headache by age and sex.

## Methods

### Study design and study population

Baseline data of the GHC were assessed between 2003 and 2005. The study was approved by the ethics committee of the University of Duisburg-Essen, Germany. Informed written consent was obtained via postal mail from all subjects. Subjects were randomly selected from statutory lists of residence drawn from three regions in Germany: the city of Essen, a large town (585,481 residents) in the region of North Rhine-Westphalia in the western part of Germany; the city of Muenster, a medium-sized town (272,890 residents), also in the western part of Germany; and Sigmaringen, a rural area, consisting of a small town with 16,501 inhabitants and 20 surrounding villages in southern Germany. Inclusion criteria were: (i) age between 18 and 65 years, and (ii) German citizenship, to ensure proper knowledge of the German language in order to properly fill out the questionnaire.

**Fig. 1:**
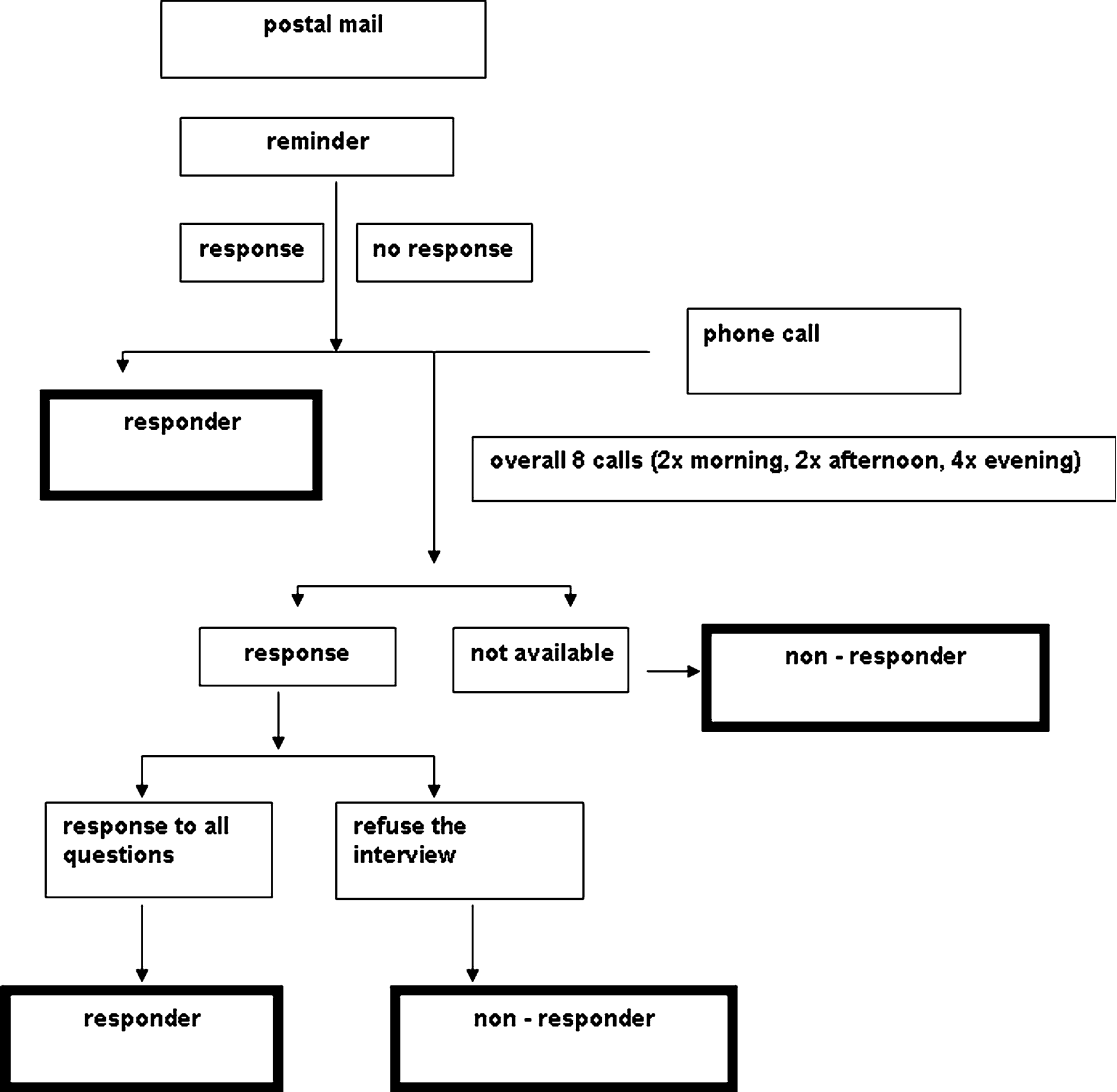
Screening procedure. Eighteen thousand randomly selected subjects received a questionnaire via postal mail. Subjects who did not respond were called for at least eight times for an interview by telephone. Main reasons for non-response by phone were that subjects were not available or refused the interview. Overall, we obtained information per mailed questionnaire or telephone interview from *n* = 9,944

**Fig. 2:**
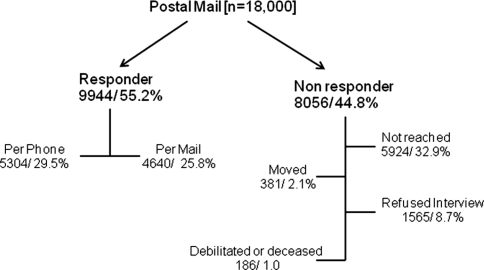
Responder versus non-responder. Overall 9,944 (55.2%) individuals responded of whom 4,640 (25.8%) answered via postal mail, and 5,304 (29.5%) by telephone. 8,056 (44.8%) were non-responder, of whom 1,565 (8.7%) refused the interview, 5,924 (32.9%) could not be reached, 381 (2.1%) moved away and 186 (1.0%) were debilitated or deceased

Figure 1 illustrates the flow chart of the screening procedure. Eighteen thousand randomly selected subjects received a questionnaire via postal mail (Fig. 1). Subjects who did not respond (44.8%) were called for at least eight times for an interview by telephone. Interviews were performed by trained medical students using the same questionnaire. Main reasons for non-response by phone were that subjects could not be reached (32.9%) or refused the interview (8.7%; Fig. 2). Overall, we obtained information per mailed questionnaire or telephone interview from *n* = 9,944 (55.2%) participants.

### Questionnaire

The questionnaire was constructed based on the criteria of the IHS [8] and validated prior to the survey in 278 patients. The results of the validation were reported elsewhere [4, 9]. First, the participants were asked if they experienced any kind of headache in the last year. The questionnaire was designed to collect demographic data, and information regarding MIG, TTH and trigeminal autonomic cephalalgias in the last 3 months. Notably, the questionnaire allowed for the diagnosis of each headache type. For example, in a person with more than one headache type, one headache type might be classified as MIG and another as TTH. The questionnaire asked a series of questions that allowed for the diagnosis of migraine and/or TTH according to the definition of the revised version of the international classification of headache disorders (ICHD-II) and the assessment of headache attack frequency per month in migraine and TTH.

The questionnaire did not allow for the diagnosis of each headache attack; this type of data is best ascertained through a daily headache diary.

### Diagnostic criteria

Definitive migraine was diagnosed if all IHS criteria were met, probable MIG if all but one IHS criteria were fulfilled, but the participant did not fulfill the IHS criteria for TTH. Definitive TTH was defined if all IHS criteria were met, probable TTH if all but one IHS criteria for TTH was met, but the participant did not fulfill the IHS criteria for MIG. Chronic daily headache was defined as headache present on ≥15 days/month and not meeting criteria for trigeminal autonomic cephalalgias (TACs). Overlap between MIG and TTH as well as either MIG or TTH and headache on ≥15 days/month was possible because the latter included chronic MIG and chronic TTH. Episodic primary headache disorders, definite MIG and definite TTH as well as probable MIG and probable TTH were summarized as episodic All-MIG (epAll-MIG) and episodic All-TTH (epAll-TTH) [10].

### Statistical analysis

The aim of the current manuscript was to provide detailed information on the prevalence of primary headaches stratified by age and gender. Therefore, all results are provided as percentages in which number of cases is divided by the number of responders who duly completed the questionnaire. Categorical variables were described as percentages with their 95% confidence intervals (95% CI). All analyses were carried out using SPSS, version 14.0 (SPSS Inc., Chicago, IL, USA).

## Results

**Fig. 3:**
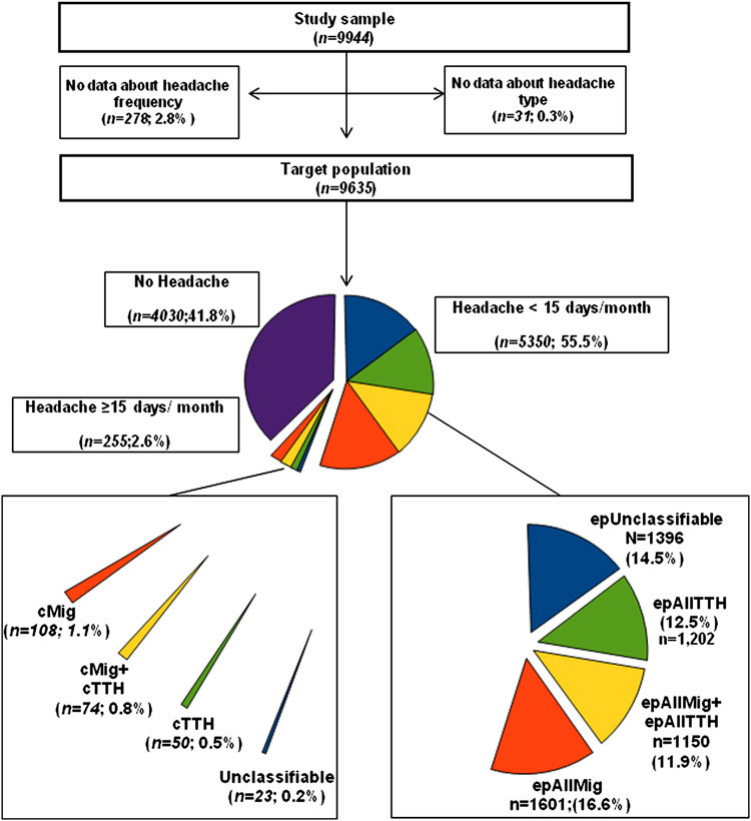
Prevalence of episodic and chronic primary headache disorders. In total, 9,944 participants responded of whom in 31 cases (0.3%) information on headache type was missing. In 278 (2.8%) cases, data about headache frequency were missing. The questionnaire was duly completed by 9,635 respondents. Episodic headache was reported by 55.5%, chronic headache by 2.6% and no headache by 41.8% of the participants

Overall 9,944 (55.2%) individuals responded (Fig. 2). Of these 4,640 (25.8%) answered via postal mail, and 5,304 (29.5%) by telephone. 8,056 (44.8%) were non-responder, of whom 1,565 (8.7%) refused the interview, 5,924 (32.9%) could not be reached, 381 (2.1%) moved away and 186 (1.0%) were debilitated or deceased. Non-responder tended to be younger (39.8 ± 12.6 resp. 43.0 ± 13.1 years) and were slightly more often men (men 53.3%, women 46.7%). Information on headache type was missing in 31 (0.3%) questionnaires (Fig. 3). These subjects only answered whether they experienced any kind of headache last year. In 278 (2.8%) cases, data about headache frequency were missing (Fig. 3).

In 9,912 cases, data about headache type were complete. The questionnaire was completed by 9,635 respondents.

### All primary headache disorders (*n* = 9,912)

Table 1 shows the prevalence of headache disorders separated into episodic and chronic headache conditions stratified by sex and age groups. The prevalence was as follows:

**Table 1:** All primary headache disorders by age and gender

All headache Age group	*N*	No headache, *n* (%) [95% CI]	All-MIG		All-MIG + TTH		All-TTH		Unclassifiable *n* (%) (95% CI)
			Prob MIG *n* (%) [95% CI]	MIG *n* (%) [95% CI]	Prob MIG + Prob TTH *n* (%) [95% CI]	MIG + TTH *n* (%) [95% CI]	Prob TTH *n* (%) [95% CI]	TTH *n* (%) [95% CI]	
*All*									
18–25	1,290	355 (27.5) [25.1–30]	75 (5.8) [4.5–7.1]	166 (12.9) [11–14.7]	134 (10.4) [8.7–12.1]	85 (6.6) [5.2–7.9]	139 (10.8) [9.1–12.5]	51 (4) [2.9–5]	285 (22.1) [19.8–24.4]
26–35	1,661	474 (28.5) [26.4–30.7]	98 (5.9) [4.8–7]	254 (15.3) [13.6–17]	181 (10.9) [9.4–12.4]	98 (5.9) [4.8–7]	168 (10.1) [8.7–11.6]	64 (3.9) [2.9–4.8]	324 (19.5) [17.6–21-4]
36–45	2,463	871 (35.4) [33.5–37.3]	103 (4.2) [3.4–5]	417 (16.9) [15.5–18.4]	198 (8) [7–9.1]	144 (5.8) [4.9–6.8]	277 (11.2) [10–12.5]	89 (3.6) [2.9–4.4]	350 (14.2) [12.8–15.6]
46–55	2,437	1,109 (45.5) [43.5–47.5]	88 (3.6) [2.9–4.4]	328 (13.5) [12.1–14.8]	170 (7) [6–8]	110 (4.5) [4–5.3]	214 (8.8) [7.7–9.9]	93 (3.8) [3.1–4.6]	322 (13.2) [11.9–14.6]
56–66	2,061	1,221 (59.2) [57.1–61.4]	69 (3.3) [2.6–4.1]	167 (8.1) [6.9–9.3]	85 (4.1) [3.3–5]	67 (3.3) [2.5–4]	160 (7.8) [6.6–8.9]	61 (3) [2.2–3.7]	239 (11.6) [10.2–13]
Total	9,912	4,030 (40.7) [39.7–41.6]	1,774 (17.9) [17.1–18.6]		1,272 (12.8) [12.2–13.5]		1,317 (13.3) [12.6–13.9]		1,520 (15.3) [14.6-16]
			433 (4.4) [14.6–16]	1,332 (13.4) [12.8–14.1]	768 (7.7) [7.2–8.3]	504 (5.1) [4.6–5.5]	958 (9.7) [9.1–10.3]	358 (3.6) [3.2–4]	
*Women*									
18–25	675	118 (17.5) [14.6–20.4]	39 (5.8) [4–7.5]	120 (17.8) 14.9–20.7]	79 (11.7) [9.3–14.1]	56 (8.3) [6.2–10.4]	86 (12.7) [10.2–15.3]	25 (3.7) [2.3–5.1]	152 (22.5) [19.4–25.7]
26–35	891	164 (18.4) [15.9–21]	62 (7.0) [5.3–8.6]	182 (20.4) [17.8–23.1]	113 (12.7) [10.5–14.9]	58 (6.5) [4.9–8.1]	104 (11.7) [9.6–13.8]	37 (4.2) [2.8–5.5]	171 (19.2) [16.6–21.8]
36–45	1,305	338 (25.9) [23.5–28.3]	67 (5.1) [3.9–6.3]	312 (23.9) [3.9–6.3]	112 (8.6) [7.1–10.1]	93 (7.1) [5.7–8.5]	155 (11.9) [10.1–13.6]	46 (3.5) [2.5–4.5]	182 (13.9) [12.1–15.8]
46–55	1,274	452 (35.5) [32.6–38.1]	52 (4.1) [3–5.2]	260 (20.4) [18.2–22.6]	95 (7.5) [6–8.9]	68 (5.3) [4.1–6.6]	114 (8.9) [7.4–10.5]	51 (4.0) [2.9–5.1]	182 (14.3) [12.4–16.2]
*56*–*66*	1,077	550 (51.1) [48.1–54.1]	33 (3.1) [2–4.1]	125 (11.6) [9.7–13.5]	55 (5.1) [3.8–6.4]	45 (4.2) [3–5.4]	98 (9.1) [7.4–10.8]	39 (3.6) [2.5–4.7]	132 (12.3) [10.3–14.2]
Total	5,222	1,622 (31.1) [29.8–32.3]	1,252 (23.9) [22.8–25.1]		774 (14.8) [13.9–15.8]		755 (14.5) [13.5–15.4]		819 (15.7%) [14.7–16.7]
			253 (4.8) [4.3–5.4]	999 (19.1) [18.1–20.2]	454 (8.7) [7.9–9.5]	320 (6.1) [5.5–6.8]	557 (10.7) [9.8–11.5]	198 (3.8) [3.3–4.3]	
*Men*									
18–25	615	237 (38.5) [34.7–42.4]	36 (5.9) [4–7.7]	46 (7.5) [5.4–9.6]	55 (8.9) [6.7–11.2]	29 (4.7) [3–6.4]	53 (8.6) [6.4–10.8]	26 (4.2) [2.6–5.8]	133 (21.6) [18.4–24.9]
26–35	770	310 (40.3) [36.8–43.7]	36 (4.7) [3.2–6.2]	72 (9.4) [7.3–11.4]	68 (8.8) [6.8–10.8]	40 (5.2) [3.6–6.7]	64 (8.3) [6.4–10.3]	27 (3.5) [2.2–4.8]	153 (19.9) [17.1–22.7]
36–45	1,158	533 (46.0) [43.2–48.9]	50 (4.3) [3.2–5.5]	105 (9.1) [7.4–10.7]	86 (7.4) [5.9–8.9]	51 (4.4) [3.2–5.6]	122 (10.5) [8.8–12.3]	43 (3.7) [2.6–4.8]	168 (14.5) [12.5–16.5]
46–55	1,163	657 (56.5) [53.9–59.6]	39 (3.4) [2.3–4.4]	68 (5.8) [4.5–7.2]	75 (6.4) [5–7.9]	42 (3.6) [2.5–4.7]	100 (8.6) [7–10.2]	42 (3.6) [2.5–4.7]	140 (12.0) [10.2–13.9]
56–66	984	671 (58.2) [65.3–71.1]	28 (2.8) [1.8–3.9]	42 (4.3) [3–5.5]	30 (3.0) [2–4.1]	22 (2.2) [1.3–3.2]	62 (6.3) [4.8–7.8]	22 (2.2) [1.3–3.2]	107 (10.9) [8.9–12.8]
Total	4,690	2,408 (51.3) [49.9–52.8]	522 (11.1) [10.2–12]		498 (10.6) [9.7–11.5]		561 (12.0) [11–12.9]		701 (14.9) [13.9–15.9]
			189 (4.0) [3.5–4.6]	333 (7.1) [6.4–7.8]	314 (6.7) [6–7.4]	184 (3.9) [3.4–4.5]	401 (8.6) [7.8–9.4]	160 (3.4) [2.9–3.9]	

*CI* confidence interval, *MIG* migraine, *prob Mig* probable migraine, *TTH* tension-type headache, *prob TTH* probable tension-type headache

All-MIG 17.9% (*n* = 1,774; [95% CI 17.1–18.6]), All-TTH 13.3% (*n* = 1,317; [95% CI 12.6–13.9]), All-MIG + TTH 12.8% (*n* = 1,272; [95% CI 12.2–13.5]), unclassifiable headache 15.3% (*n* = 1,520; [95% CI 14.6–16.0]) (Table 1). In women, All-MIG (23.9%; [95% CI 22.8–25.1]) and unclassifiable headache were most frequent (15.7%; [95% CI 14.7–16.7]). All-MIG + TTH and All-TTH were quite similar (Table 1). The prevalence among men was as follows: All-MIG 11.1% (*n* = 522; [95% CI 10.2–12.0]), All-TTH 12.0% (*n* = 561; [95% CI 11.0–12.9]), All-MIG + TTH 10.6% (*n* = 498; [95% CI 9.7–11.5]), unclassifiable headache 14.9% (*n* = 701; [95% CI 13.9–15.9]). It is of note that participants with migraine were identified to suffer more from definite migraine than probable migraine (3.1-fold), whereas in tension-type headache and MIG + TTH subjects tend to suffer more probable TTH and probable MIG + TTH (2.7- and 1.5-fold).

### Episodic headache (*N* = 9,635)

Figure 3 depicts the overall prevalence of headache in 9,635 participants followed by the distribution of headache type, out of which 55.5% reported headache that occurred on less than 15 days per month. Table 2 shows the prevalence of episodic headache types with regards to sex and age group. Of those suffering from headache, episodic All-MIG was the most common reported headache type with 29.9% (*n* = 1,601; [95% CI 28.7–31.2]), followed by unclassifiable episodic headache 26.1% (*n* = 1,396; [95% CI 24.9–27.3]), epAll-TTH 22.5% (*n* = 1,202; [95% CI 21.4–23.6]), and epAll-MIG + TTH 21.5% (*n* = 1,150; [95% CI 20.4–22.6]).

**Table 2:** Episodic primary headache disorder

epHeadache age group	*N*	epAllMIG *n* (%) [95% CI]	epAllMIG + TTH *n* (%) [95% CI]	epAllTTH *n* (%) [95% CI]	epUnclassifiable *n* (%) [95% CI]
*All*					
18–25	854	221 (25.9) [22.9–28.8]	194 (22.7) [19.9–25.5]	177 (20.7) [18–23.4]	262 (30.7) [27.6–33.8]
26–35	1,116	328 (29.4) [26.7–32.1]	262 (23.5) [21–26]	218 (19.5) [17.2–21.9]	308 (27.6) [25–30.2]
36–45	1,482	498 (33.6) [31.2–36]	318 (21.5) [19.4–23.6]	344 (23.2) [21.1–25.4]	322 (21.7) [19.6–23.8]
46–55	1,190	367 (30.8) [28.2–33.5]	249 (20.9) [18.6–23.2]	279 (23.4) [21–25.9]	295 (24.8) [22.3–27.2]
56–66	707	187 (26.4) [23.2–29.7]	127 (18) [15.1–20.8]	184 (26) [22.8–29.3]	209 (29.6) [26.2–32.9]
Total	5,349	1,601 (29.9) [28.7–31.2]	1,150 (21.5) [20.4–22.6]	1,202 (22.5) [21.4–23.6]	1,396 (26.1) [24.9–27.3]
*Women*					
18–25	507	145 (28.6) [24.7–32.5]	119 (23.5) [19.8–27.2]	102 (20.1) [16.6–23.6]	141 (27.8) [23.9–31.7]
26–35	685	229 (33.4) [29.9–37]	158 (23.1) [19.9–26.2]	134 (19.6) [16.6–22.5]	164 (23.9) [20.8–27.4]
36–45	904	353 (39) [35.9–42.2]	192 (21.2) [18.6–23.9]	189 (20.9)	170 (18.8)
				[18.3–23.6]	[16.3–21.4]
46–55	747	274 (36.7) [33.2–40.1]	144 (19.3) [16.5–22.1]	156 (20.9) [18–23.8]	173 (23.2) [20.1–26.2]
56–66	453	134 (29.6) [24.4–33.8]	84 (18.5) [15–22.1]	116 (25.6) [21.6–29.6]	119 (26.3) [22.2–30.3]
Total	3,296	1,135 (34.4) [32.8–36.1]	697 (21.1) [19.8–22.5]	697 (21.1) [19.8–22.5]	767 (23.3) [21.8–24.7]
*Men*					
18–25	347	76 (21.9) [17.6–26.3]	75 (21.6) [17.3–25.9]	75 (21.6) [17.3–25.9]	121 (34.9) [29.9–39.9]
26–35	431	99 (23) [19–26.9]	104 (24.1) [20.1–28.2]	84 (19.5) [15.8–23.2]	144 (33.4) [29–37.9]
36–45	578	145 (25.1) [21.6–28.6]	126 (21.8) [18.4–25.2]	155 (26.8) [23.2–30.4]	152 (26.3) [22.7–29.9]
46–55	443	93 (21) [17.2–24.8]	105 (23.7) [19.7–27.7]	123 (27.8) [23.6–31.9]	122 (27.5) [23.4–31.7]
56–66	254	53 (20.9) [15.9–25.9]	43 (16.9) [12.3–21.5]	68 (26.8) [21.3–32.2]	90 (35.4) [29.6–41.3]
Total	2,053	466 (22.7) [20.9–24.5]	453 (22.1) [20.3–23.9]	505 (24.6) [22.7–26.5]	629 (30.6) [28.6–32.6]

*CI* confidence interval, *epAllMIG* episodic all migraine, *epAllTTH* episodic all tension-type headache, *epUnclassifiable* episodic unclassifiable, *epAllMIG* *+* *TTH* episodic all migraine + tension-type headache, *epHeadache* episodic headache

Among women epAll-MIG was the most frequent (34.4%, 32.8–36.1), whereas all other headache types were almost equally distributed. In men, unclassifiable headache was 30.6% (*n* = 629; [95% CI 28.6–32.6]), followed by epAll-TTH 24.6% (*n* = 505; [95% CI 22.7–26.5]), epAll-MIG 22.7% (*n* = 466; [95% CI 20.9–24.5]) and epAll-MIG + TTH 22.1% (*n* = 453; [95% CI 20.3–23.9]). Compared to men, women reported 2.4-fold increase of epAll-MIG, 1.4 of epAll-TTH, and 1.5 of epAll-MIG + TTH.

In relation to age, Table 2 shows that in women epAll-MIG peaks between 36 and 45 years and epAll-TTH and epAll-MIG + TTH between 18 and 35 years. In men, epAll-MIG peaks between 36 and 45 years, epAll-TTH show similar frequency in the age groups between 36 and 66 years and epAll-MIG + TTH show two peaks in the age groups 26 and 35 years and 46 and 55 years (Table 2).

### Chronic headache

We identified 255 participants that reported headache on at least 15 days/month resulting in a prevalence of 2.6% (95% CI 2.3–3) (Table 3). The prevalence was as follows: chronic MIG 42.4% (*n* = 108; [95% CI 36.3–48.4]), chronic MIG + TTH 29% (*n* = 74; [95% CI 23.5–34.6]), chronic TTH 19.6% (*n* = 50; [95% CI 14.7–24.5]) and unclassifiable headache 9% (*n* = 23; [95% CI 5.5–12.5]). Women were more frequently affected than men (65.1 vs. 34.9%). Apparently chronic headache in women tends to become a bigger problem with increasing age (Table 3). In particular, chronic MIG and chronic MIG + TTH in women peak between 36 and 55 years, whereas chronic TTH peaks in the age group 56 and 66 years. In men chronic MIG peaks between 26 and 35 years, chronic MIG + TTH between 18 and 25 years and chronic TTH between 46 and 55 years.

**Table 3:** Chronic headache

CH age groups	*N*	cMIG *n* (%) [95% CI]	cMIG + TTH *n* (%) [95% CI]	cTTH *n* (%) [95% CI]	cUnclassifiable *n* (%) [95% CI]
*All*					
18–25	36	13 (36.1) [20.4–51.8]	13 (36.1) [20.4–51.8]	5 (13.9) [2.6–25.2]	5 (13.9) [2.6–25.2]
26–35	27	12 (44.4) [25.7–63.2]	9 (33.3) [15.6–51.1]	4 (14.8) [1.4–28.2]	2 (7.4) [0–17.3]
36–45	44	19 (43.2) [28.6–57.8]	13 (29.5) [16.1–43]	8 (18.2) [6.8–29.6]	4 (9.1) [0.6–17.6]
46–55	68	35 (51.5) [39.6–63.4]	29 (42.6) [30.9–54.4]	10 (14.7) [6.3–23.1]	3 (4.4) [0–9.3]
56–66	80	29 (36.3) [25.7–46.8]	19 (23.7) [14.4–33.1]	23 (28.8) [18.8–38.7]	9 (11.3) [4.3–18.2]
Total	255	108 (42.4) [36.3–48.4]	74 (29) [23.5–34.6]	50 (19.6) [14.7–24.5]	23 (9) [5.5–12.5]
*Women*					
18–25	30	12 (40) [22.5–57.5]	10 (33.3) [16.5–50.2]	4 (13.3) [1.2–25.5]	4 (13.3) [1.2–25.5]
26–35	20	8 (40) [18.5–61.5]	8 (40) [18.5–61.5]	3 (15) [0–30.7]	1 (5) [0–14.6]
36–45	26	14 (53.8) [34.7–73]	8 (30.8) [13–48.5]	3 (11.5) [0–23.8]	1 (3.8) [0–11.2]
46–55	46	28 (60.9) [46.8–75]	16 (34.8) [21–48.6]	1 (2.2) [0–6.4]	1 (2.2) [0–6.4]
56–66	44	17 (38.6) [24.3–53]	11 (25) [12.2–37.8]	13 (29.5) [16.1–43]	3 (6.8) [0–14.3]
Total	166	79 (47.6) [40–55.2]	53 (31.9) [24.8–39]	24 (14.5) [9.1–19.8]	10 (6) [2.4–9.6]
*Men*					
18–25	6	1 (16.7) [0–46.5]	3 (50) [10–90]	1 (16.7) [0–46.5]	1 (16.7) [0–46.5]
26–35	7	4 (57.1) [20.5–93.8]	1 (14.3) [0–40.2]	1 (14.3) [0–40.2]	1 (14.3) [0–40.2]
36–45	18	5 (27.8) [7.1–48.5]	5 (27.8) [7.1–48.5]	5 (27.8) [7.1–48.5]	3 (16.7) [0–33.9]
46–55	22	7 (31.8) [12.4–51.3]	4 (18.2) [2.1–34.3]	9 (40.9) [20.4–61.5]	2 (9.1) [0–21.1]
56–66	36	12 (33.3) [17.9–48.7]	8 (22.2) [8.6–35.8]	10 (27.8) [13.2–42.4]	6 (16.7) [4.5–28.8]
Total	89	29 (32.6) [22.9–42.3]	21 (23.6) [14.8–32.4]	26 (29.2) [19.8–38.7]	13 (14.6) [7.3–21.9]

*CI* confidence interval, *CH* chronic headache, *cMIG* chronic migraine, *cTTH* chronic tension-type headache, *cUnclassifiable* chronic unclassifiable

## Discussion

Population-based surveys assessing the prevalence of primary headache disorders in Germany are rare. The aim of our present work was to provide prevalence data of primary headache disorders including episodic and chronic headache forms in relation to sex and age in the general population in Germany. About 55% of the participants reported any kind of headache within the last 12 months.

As far as migraine is concerned, our results are comparable to previous studies from other European countries and the USA [11–16]. The prevalence of migraine in Sweden was 13.2, 16.7% among women and 9.5% among men [11], in England 18.3% among women and 7.6% among men [15] compared to the 13.4% of migraine prevalence in our study, 19.1% among women and 7.1% among men. The estimated prevalence of definite MIG and probable MIG in France and Austria was similar: 11.2% for definite MIG and 10.1% for probable MIG in France [13], 10.2% for definite MIG and 8.5% for probable MIG in Austria [12] compared to 13.4% definite MIG and 4.4% probable MIG in the present study. In the USA, the prevalence of MIG is about 17% in women and 6% in men [14, 16] which is comparable to our data as well (19.1% in women and 7.1% in men).

Previous data on the prevalence of TTH are more heterogeneous. In Germany, the lifetime prevalence of TTH was reported to be 38.5% [6]. The 1-year prevalence in Croatia was 20.7% [17]. In Denmark, the 1-year prevalence of TTH in men was found to be 63 and 86% in women [18]. Several reasons may lead to this wide variation of estimated prevalence in TTH. For example, questionnaires may differ in their sensitivity to identify cases where respondents consider their symptoms trivial. The estimated 1-year prevalence of episodic TTH in our survey is comparable to estimates in Croatia [17] but is lower than previous estimates in Germany [6], in Georgia [10] and in Denmark [18].

We found 1,392 subjects with a headache frequency <15 days/month but with missing data to allow for further headache classification. Trigeminal autonomic cephalalgias (TAC) and other primary headache disorders are unlikely to be detected in a population-based setting since the prevalence of these headache syndromes is rather low. Patients with TACs might be easily misclassified as either MIG or TTH leading to diverse results in either direction dependent on the design and focus of the respective questionnaire. As migraine is diagnosed essentially on the presence of specific features, and TTH by the absence of those same features, we think that these cases were most probably classified as TTH rather than migraine by our questionnaire.

In our population, the overall 1-year prevalence of chronic headache was 2.6%. These results are in line with studies from Western Europe and the USA [19–21]. In Western Europe, chronic headache was reported to be 4.7% in participants aged over 14 years [19] out of which chronic MIG was found to be 2.4% and chronic TTH 2.2%. In a large population-based survey from France, the overall prevalence of chronic headache in subjects aged over 15 years was 2.9% out of which two-thirds had chronic migraine [20]. In the USA, the overall prevalence of chronic headache between 18 and 65 years was 4.1% (female to male ratio: 1.8:1). Interestingly, the subjects in this study more often reported chronic TTH (>50%) than chronic MIG (female: 33%, male 25%) [21]. Chronic headache that could not be clearly identified was found in 15% in women and 19% in men [21]. Subjects that reported headache on 15 days/month or more in our study, chronic MIG was more frequent (42.4, 47.6% in women and 32.6% in men) than chronic TTH (19.6, 14.5% in women and 29.2% in men). The women to men ratio was 2.7:1 for chronic MIG and 0.9:1 for chronic TTH.

The strengths of our study are the large population-based sample recruited in 3 different regions of Germany, the high response rate of 55%, and the use of a validated questionnaire. A false negative rate around 15% is compatible with the sensitivities of our questionnaire for MIG (0.73) and TTH (0.85) [4, 9].

Some limitations of our study need to be addressed. Although the response of our survey was satisfactory, possible selection bias might have occurred. For example, very old and very young people more often refuse to take part in surveys, and persons that suffer from headache possibly more often participate in survey seemingly affecting them, than persons not suffering from headache at all. However, these limitations pose a challenge that all questionnaire-based surveys have to face.

In conclusion, we provided comprehensive and detailed prevalence estimates of primary headache disorders in the general population in Germany. These data are valuable in emphasizing the importance of episodic and chronic headache in regard to health economics and the burden of disease within a population. The next steps will have to include the in-depth investigation of associated risk factors that may be associated with episodic headache and lead to chronic headache. There is much more to learn about the role of possible influencing factors like socioeconomic status, smoking, or obesity on headache prevalence in the general population.
